# RETRACTION: The Antitumor Activity of *Antrodia camphorata* in Melanoma Cells: Modulation of Wnt/*β*‐Catenin Signaling Pathways

**DOI:** 10.1155/ecam/9802978

**Published:** 2025-12-07

**Authors:** Evidence-Based Complementary and Alternative Medicine

RETRACTION: Y‐C. Hseu, H‐T. Tsou, K. J. S. Kumar, K‐Y. Lin, H‐W. Chang, and H‐L. Yang, “The Antitumor Activity of *Antrodia camphorata* in Melanoma Cells: Modulation of Wnt/*β*‐Catenin Signaling Pathways,” *Evidence-Based Complementary and Alternative Medicine* 2012, no. 1 (2012): 1–14, https://doi.org/10.1155/2012/197309.

The above article, published online on 25 September 2012 in Wiley Online Library (https://wileyonlinelibrary.com), has been retracted by agreement between the journal Editor, Jian‐Li Gao and John Wiley & Sons Ltd.

The retraction has been agreed upon following an investigation into concerns raised by a third party and Pubpeer comments [1] regarding image inconsistencies. Reused images were identified in Figures 2a, 3a, 4b, 5b, 6b, and 6c, whilst claiming to be different. The authors’ responses regarding these findings were insufficient.

• Figure 2a reused the same bands for B16F10 *β*‐actin and Figure 3a B16F10 Histone
• Figure 3a reused the same *β*‐actin bands for B16F10 and B16F1
• Figure 4b reused the same *β*‐actin bands for B16F10 and Figure 5a B16F10
• Figure 5b DAPI/0 µg/mL is reused in a later article by the authors [2], Figure 4A DAPI/0 µM
• Figure 6b reused the same image for B16F10 and B16F1 20 µg/mL
• Figure 6c reused the same *β*‐actin bands for B16F10 and B16F1

The authors disagree with the retraction.
